# Calibration and XGBoost reweighting to reduce coverage and non-response biases in overlapping panel surveys: application to the Healthcare and Social Survey

**DOI:** 10.1186/s12874-024-02171-z

**Published:** 2024-02-15

**Authors:** Luis Castro, María del Mar Rueda, Carmen Sánchez-Cantalejo, Ramón Ferri, Andrés Cabrera-León

**Affiliations:** 1 Department of Statistics and Operational Research, University of Granada, Granada, Spain; 2 Institute of Mathematics, University of Granada, Granada, Spain; 3 Department of Public Health, Andalusian School of Public Health, Granada, Spain; 4 Centro de Investigación Biomédica en Red de Epidemiología y Salud Pública (CIBERESP), Instituto de Salud Carlos III, Madrid, Spain

**Keywords:** Public health, COVID-19, Panel surveys, Sampling, Machine learning, Non-response bias

## Abstract

**Background:**

Surveys have been used worldwide to provide information on the COVID-19 pandemic impact so as to prepare and deliver an effective Public Health response. Overlapping panel surveys allow longitudinal estimates and more accurate cross-sectional estimates to be obtained thanks to the larger sample size. However, the problem of non-response is particularly aggravated in the case of panel surveys due to population fatigue with repeated surveys.

**Objective:**

To develop a new reweighting method for overlapping panel surveys affected by non-response.

**Methods:**

We chose the Healthcare and Social Survey which has an overlapping panel survey design with measurements throughout 2020 and 2021, and random samplings stratified by province and degree of urbanization. Each measurement comprises two samples: a longitudinal sample taken from previous measurements and a new sample taken at each measurement.

**Results:**

Our reweighting methodological approach is the result of a two-step process: the original sampling design weights are corrected by modelling non-response with respect to the longitudinal sample obtained in a previous measurement using machine learning techniques, followed by calibration using the auxiliary information available at the population level. It is applied to the estimation of totals, proportions, ratios, and differences between measurements, and to gender gaps in the variable of self-perceived general health.

**Conclusion:**

The proposed method produces suitable estimators for both cross-sectional and longitudinal samples. For addressing future health crises such as COVID-19, it is therefore necessary to reduce potential coverage and non-response biases in surveys by means of utilizing reweighting techniques as proposed in this study.

**Supplementary Information:**

The online version contains supplementary material available at 10.1186/s12874-024-02171-z.

## Background

Healthcare statistical services worldwide have used probability surveys to provide information on the social, economic and health impact of the disease, or on its seroprevalence [1] and evolution, or on the characteristics of the infected population, in particular members most vulnerable to the virus due to their age, risk of exclusion, health conditions or dependency [2]. These surveys allow valid inferences to be made about the population without having to incorporate hypotheses into the models, which is of great practical benefit [3]. Regarding the COVID-19 pandemic, most of the surveys created were based on non-probability sampling to provide a quick and efficient assessment of the situation based on predicting and quantifying the main parameters involved in this phenomenon [4].

The Healthcare and Social Survey (ESSA, Encuesta Sanitaria y Social de Andalucía) research project arises from the need to provide data on the evolution of the COVID-19 impact which can be considered when making decisions to prepare and deliver an effective Public Health response in the different populations concerned, particularly in the most vulnerable ones, including, for example, the elderly, the chronically ill, or persons at risk of exclusion [5]. The objective of this survey is to determine the magnitude, characteristics, and evolution of the impact of COVID-19 on overall health and its socioeconomic, psychosocial, behavioral, occupational, environmental, and clinical determinants in the general population and in the population at higher risk for socioeconomic deprivation. The study is based on a Real-World Data design integrating observational data extracted from multiple sources including information obtained from different surveys and clinical, population, and environmental registries. The ESSA has an overlapping panel design [6]. It consists of a series of measurements broken down into a new sample and a longitudinal sample for each measurement, except for the first measurement where the entire sample is new. Compared to rotating panel surveys [7], the ESSA sampling design is therefore non-rotational, i.e. the units included in each measurement remain in the following measurements until the final one.

This type of overlapping panel design is often used when the main objectives are to obtain cross-sectional estimates at time *t* and short-term longitudinal estimates of net and gross change between *t* and $t+1$, as is the case for ESSA. This way, the use of new samples at each measurement *t* permits whole population representativeness at time $t+1$, and therefore also permits cross-sectional estimation at this time. This feature means that one of the key aspects of overlapping panel surveys lies in cross-sectional estimation, i.e. how to combine the different samples selected at the same time. Another key aspect of panel surveys is the response obtained in each measurement of the longitudinal samples. The lack of response thus grows with the number of occasions or measurements, due, amongst other reasons, to the panelist fatigue with repeated interview. For this reason, partial replacement of units is common to guarantee a minimum number of units in the final sample. Estimation from data obtained with this structure is not easy, especially if the desire is to take into account the biases produced both by lack of response and lack of sample coverage and representativeness.

Some methods of handling wave non-response in panels are provided in [8–10]. Thus, the two main methods used to handle it in panels are based on weighting the effective sample according to the theoretical sample in the strata used, and reweighting by calibration in terms of population totals for sociodemographic stratification variables such as sex, age or territory (e.g. region, province or habitat level) [11]. Another set of studies focuses on modelling different types of response patterns in panels. Kern et al. [12] compares the usage of different Machine Learning (ML) methods for modeling non-response in the German Socio-Economic Panel Study (GSOEP) and recent study [13] proposes a general framework for building and evaluating non-response prediction models with panel data, although this study focuses on model building and evaluation without utilizing the predictions obtained to correct bias in the estimations.

Non-response in panel studies has traditionally been tackled by using non-response weights. Although reweighting methods do exist for addressing these types of biases, they have been proposed fundamentally for cross-sectional surveys and there are few studies that provide a formal methodology for their treatment in this type of panel survey. In [14], the authors discuss adjustments for non-response and how calibration can be carried out in panel studies in general and what effects it creates. They consider three possible calibration approaches: initial calibration (at the beginning of the panel, the weights of the units in the panel are calibrated), final calibration (at measurement *t* the weights of the individuals in the sample are adjusted by calibration) and initial and subsequent final calibration (both initial and final calibration are carried out). Several approaches are tested in [15] to produce calibration estimators which are suitable for survey data affected by non response where auxiliary information exists at both the panel and population level.

Longitudinal and cross-sectional weighting are considered in [7] for rotating samples in the context of the SILC survey in France. The sampling each year in this survey is formed by combining nine panel subsamples, and the longitudinal weights are allocated as an average of the weights in each time during which a unit belongs to the sample, using the weight-share method [16]. This method is also used in [17] for obtaining cross-sectional indicators for the SILC survey in Switzerland based on a four-panel rotation scheme. Verma et al. [18] develops longitudinal and cross-sectional weighting procedures in a rotational household panel with reference to the EU-SILC design (4-year rotational design) using a step-by-step procedure starting with design weights, followed by adjustments for non-response and calibration to external controls, and finally trimming and scaling as required to obtain the initial weights.

However, these authors do not consider the application of these adjustment methods in designs such as the study described herein, i.e. overlapping panel surveys where the units included in each measurement remain in the following measurements until the final one, and in which each measurement is completed with a new sample (except for the first one since the whole sample is new). This is the research gap addressed by this paper.

In this work, we therefore propose an empirical study of the associations between choice of research methodology and study outcomes. Accordingly, we combine suitable reweighting methods such as Propensity Score Adjustment (PSA), XGBoost and calibration to address the biases associated with dropout from overlapping panel survey data for estimating totals, proportions, ratios and differences in a study outcome. Other ML methods than XGBoost technique (such as logistic regression, decision trees, random forests and so on) could be used, but several papers [13, 19, 20] show that the set of predictor variables used in general mattered more than the type of ML technique. With regard to neural networks, they have been hugely successful for image, text or audio data due to the use of structures far more advanced than deep feedforward networks. However, for tabular data as in our case, the inefficacy and unreliability of neural networks is widely known. Arik and Pfister [21] further explains this issue in its introduction. Thus, those statistical techniques (PSA, XGBoost and calibration) are formulated on the outcome self-perceived general health from the ESSA survey and can be applied to any other variable and epidemiological research based on overlapping panel design.

## Methods

### The ESSA study framework

The Healthcare and Social Survey (ESSA, Encuesta Sanitaria y Social de Andalucía) provides a follow-up over time of the impact of the pandemic and its resulting lockdown on the population of Andalusia over the age of 16. Andalusia is a southern region of Spain with 8.4 million inhabitants. It is also the fifth most populated region in Europe, with a population similar in size to that of other European countries such as Austria or Switzerland.

**Fig. 1:**
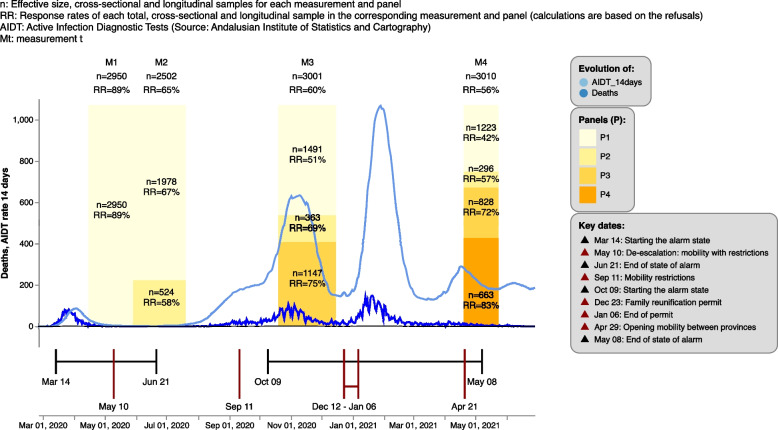
Temporal scope, response rates (RR) and effective sample size for each measurement in ESSA

As shown in Fig. 1, the ESSA study includes four measurements. The first one, $M_1$, coincided with the beginning of the Spanish State of Alarm in April 2020 (coinciding with the lockdown), while the second measurement $M_2$ was taken in June and July (a month after the first interview, coinciding with the de-escalation); the third measurement $M_3$ was taken in November and December (6 months after the first interview and coinciding with the second wave of the pandemic), and the fourth measurement $M_4$ in April and May 2021 (12 months after the first interview, coinciding with the relaxation of mobility restrictions and the end of the state of alarm). All the measurements had an effective size of around 3000 people, except for the second one which was 2500. They were obtained using an overlapping panel design, so the individuals from the previous measurement are sampled again. Each measurement thus had its own panel of people who were interviewed again in the following measurements ($P_1,...,P_4$). Non-response was offset in each measurement with another sample which included new individuals. The details of this non-response and the effective sample size for each measurement and panel can be consulted in Fig. 1. It also provides a description of the evolution of the SARS-COV-2 pandemic in Andalusia during 2020 and 2021 in terms of active infection diagnostic tests and deaths.

With respect to the sampling method, the new sample in each measurement was selected by stratified simple random sampling according to province and degree of urbanization: urban, semi-urban and rural, based on the methodology described by EUROSTAT for the allocation of territorial typologies in statistical grids of 1 $km^{2}$ where population resides; more information in [22]. This implies that within each stratum any person has the same probability of being selected, i.e. self-weighted samples are obtained in each stratum. The new sample was thus distributed across the 8 Andalusian provinces in proportion to province population size. Within each province, sample allocation was proportional to the population size of each degree of urbanization. Regarding for the longitudinal sample of a given measurement, this comprised the samples in the previous measurements, i.e. of the panel created in each measurement ($P_1,...,P_4$), with the exception of the first measurement, which did not have a longitudinal sample given that it was the first one. The population framework used for the extraction of population samples aged 16 years old and over residing in family dwellings in Andalucía, came from the Longitudinal Population Database of Andalusia (BDLPA) as of 1 January 2019. The BDLPA originates from integrating data obtained from the Civil Registries with respect to births, deaths, and marriages (i.e., vital statistics), as well as that reported in the population and housing censuses, give rise to an integrated longitudinal frame for population and territorial statistics in Andalusia [23]. The Andalusian Institute of Statistics and Cartography (IECA, Spanish acronym) was responsible for population framework and extraction of the samples. A detailed description of the protocol followed for this survey can be seen in [5].

$$\begin{aligned} \begin{array}{l} s^{(1)}_{rh} = \{ k \ \in \ s^{(1)} / \text {respond in stratum h}\}\\ s^{(1)}_{fh}=\{ k \ \in \ s^{(1)} / \text {missing in stratum h} \}. \end{array} \end{aligned}$$

### Sampling setup in overlapping panels

Let *U* denote a finite population of size *N*, $U = \left\{ 1,\ldots ,k,\ldots ,N\right\}$. We want to estimate a population parameter of a variable of interest, *y*.

On the first measurement (*M*1) a theoretical sample $s^{(1)}$ of size $n^{(1)}$ is selected from the population *U* by stratified simple random sampling. Let *h* be the stratum to which unit *i* belongs, $(h=1,...L)$ and $s^{(1)}_h$ be the sample corresponding to stratum *h* on measurement 1 as well as the first panel of the survey (*P*1).

There is a total lack of response in the sample $s^{(1)}$ which is divided into

Let $n^{(1)}_{rh}$ denote the number of the observations obtained from the $n^{(1)}_h$ sampled units, that is $\sum _h n^{(1)}_{rh} = n^{(1)}_{r}$ is the effective size of $s^{(1)}_r$ as well as of $P_1$. Thus, $s^{(1)}_r$ will be the theoretical sample of the second measurement $M_2$, $s^{(1,2)}_r$ the effective sample of $M_2$ (respondents in $M_1$ and $M_2$ of $P_1$), and $n^{(1,2)}_{r}$ the effective size of $P_1$ in $M_2$.

In each of the following measurements until *t*, $M_2,...,M_t$, we denote by $s^{(1,t)}_{r}$ the effective sample in $M_t$ of $P_1$, i.e. respondents in $M_1$, $M_2$,...,$M_t$ of the first sample obtained $s^{(1)}$ as well as of the first panel created $P_1$; and by $n^{(1,t)}_{r}$ its effective size. In a similar way, we denote by $s^{(i,j)}_{r}$, where $i=1,...,j-1$, $j=2,...,t$, and $i<j$, the effective sample in $M_j$ of $P_i$, i.e. respondents in $M_i$, $M_{i+1}$,...,$M_j$ of the theoretical sample obtained in $M_i$, $s^{(i)}$, as well as of the i panel created, $P_i$; and by $n^{(i,j)}_{r}$ its effective size. By contrast, we denote by $s^{(i,j)}_{f}$ the missing sample in $M_j$ of $P_i$, i.e. non-respondents in any $M_i$, $M_{i+1}$,...,$M_j$ of the i theoretical sample obtained in $M_i$, $s^{(i)}$, which created the i panel, $P_i$. Thus, due to this non-response sample and in order to achieve the required sample size, we complete the sample of $M_j$ with a new theoretical sample $s^{(j)}$ from the same population *U* by the same sampling design but independently of the new samples extracted in previous measurements. Therefore, for the extraction of the new theoretical samples, $s^{(1)},...,s^{(t)}$, in each new measurement $M_j$, IECA verified that $M_j$ and $M_{j-1}$ had an empty intersection. Therefore, the theoretical sample of $M_j$ comprises by the effective samples of $M_{j-1}$ and the new theoretical sample of $M_j$, i.e. $s^{M_j} = s_r^{M_{j-1}} \cup s^{(j)}$; while the effective sample of $M_j$ comprises the effective samples of the panels created until $M_{j-1}$, $P_1, ..., P_{j-1}$, and the new effective sample of $M_j$, i.e. $s_r^{M_j} = \cup _{i=1}^{j-1}s_r^{(i,j)} \cup s_r^{(j)}$.

With respect to sample size, let $n^{(j)}$ be the theoretical size of the new theoretical sample $s^{(j)}$ in measurement *j*, and denote by $n^{(j)}_r$ the effective size of $s^{(j)}_r$, i.e. the respondents of the new sample in that measurement. Thus, the theoretical size of $M_j$ comprises the effective sizes of $M_{j-1}$ and the new theoretical size of $M_j$, i.e. $n^{M_j} = n_r^{M_{j-1}} + n^{(j)}$; while the effective size of $M_j$ is composed by the effective sizes of the panels created until $M_{j-1}$, $P_1, ..., P_{j-1}$, and by the new effective size of $M_j$, i.e. $n_r^{M_j} = \sum _{i=1}^{j-1}n_r^{(i,j)} + n_r^{(j)}$.

Let $y_i^{(1)}$ be the value of the target variable associated to the *i*-th unit in $M_1$, and let $d_i$ be the design weight associated to the *i*-th unit equal to the inverse of the inclusion probability in the theoretical sample, an estimation of the total of Y in the first measurement is given by [24]:

1 $$\begin{aligned} \hat{Y}_{s^{(1)}} = \sum _{i \in s^{(1)}_{r}} d_{i} y_{i}^{(1)} \,. \end{aligned}$$

This estimator is a naive estimator. In the case of simple random sampling design for unit *i* is $d_{i}= \frac{N}{n^{(1)}}$.

Design weights should be adjusted to consider non-response in order to reduce the possible bias of resulting estimates, which may arise when there is a different propensity in answering for different groups. In the first measurement a response rate is determined in each class and a new weight is defined as the product of the design weight and the inverse of the response rate. The response rate is evaluated as $r^{(1)}= \frac{n^{(1)}_{r}}{n^{(1)}}$. Then the initial weight of unit *i* is replaced with the new weight $d_{i}^{(1)}= \frac{d_{i}}{r^{(1)}}$ and the estimator is given by

2 $$\begin{aligned} \hat{Y}_{s^{(1)}_r}= \sum _{i \in s^{(1)}_{r}} d_{i}^{(1)} y_{i}^{(1)} \,. \end{aligned}$$

For the following measurements until *t*, different estimators can be obtained from the different effective samples of measurements and panels. Thus, to fix the notation, we will term cross-sectional estimator of a parameter $\theta$ at time *j* as being those estimators that are obtained from the effective sample of $M_j$, i.e. $s_r^{M_j}$, where $j=1,...,t$; while we will call longitudinal estimators of a parameter $\theta$ at times *j* and $j-1$, to those obtained from the effective samples of those panels which belong to two consecutive measurements $M_j$ and $M_{j-1}$, i.e. $s_r^{(M_j,M_{j-1})} = \cup _{i=1}^{j-1}s_r^{(i,j)}$, where $j=2,...,t$; being the same sample as $s_{cross}^{(j)}$ but without the new effective sample of $M_j$. The process for obtaining them is shown in the following sections.

### Cross-sectional estimation

The objective of most cross-sectional surveys is to produce unbiased estimates of totals or means at a given point in time, and, in the case of repeated surveys, to produce estimates of the net change that occurred in the population between two time points [25].

Cross-sectional estimates can be derived from longitudinal survey data to improve the cost-effectiveness of surveys, assuming that the survey design takes this possibility into account, and that estimation procedures are developed to satisfy cross-sectional as well as longitudinal requirements [26]. For this we will use both the longitudinal samples from the panels and the fresh or new samples obtained in each measurement. This way, the sample we work with always has the maximum sample size possible and we reduce the final estimator variance.

Point estimation of parameters of the cross-sectional population based on data from longitudinal surveys has been studied by [27] among others and the problem of formal comparison of the estimates from two years, which requires variance estimation for the difference of the estimates, is considered in [28]. We will follow community-agreed standards appropriate for the survey methodology used in those works, but implementing a new approach to achieve more suitable estimators in overlapping panel surveys. We will thus devise a cross-sectional weighting scheme that includes a non-response adjustment, optimal combination of the samples from the panels involved, and calibration for completing the representativeness of the population at a given measurement. This proposal is described below.

#### Weight adjustment based on propensities

A simple adjusted estimator accounting for initial non-response and attrition can be obtained by adjusting the basic weights of the Horvitz-Thompson estimator by the fraction of non-response. This adjustment based on weighting within classes assumes that unit non-response may be modelled by response homogeneity groups, and that these response homogeneity groups are given by the strata. This may be a reasonable assumption at baseline but it seems unlikely that non-response at any point in time will be suitably explained by the strata defined at baseline.

Therefore, although weighting within classes is a commonly used procedure for non-response cross-sectional and longitudinal weighting in panels, a more pragmatic alternative is to use a regression-based approach, all the more so when numerous auxiliary variables are available [18]. For this we are going to use the popular Propensity Score Adjustment (PSA) method [20, 29, 30] to model the probability that a unit *k* of the new theoretical sample $s^{(j)}$ responds to $M_j$, where $j=1,...,t$, or that another unit *k* of the effective sample $s^{(i)}_r$ responds to $M_j$, where $i=1,...,j-1$, $j=2,...,t$, and $i<j$.

For each sample unit *k* in $s^{(j)}$ let be $\delta ^{(j)}_k =1$ if $k \in s^{(j)}_r$ and $\delta ^{(j)}_k= 0$ if $k \in s^{(j)} - s^{(j)}_r$, and regarding each sample unit *k* in $s^{(i)}_r$ let be $\delta ^{(i,j)}_k =1$ if $k \in s^{(i,j)}_r$ and $\delta ^{(i,j)}_k= 0$ if $k \in s^{(i)}_r- s^{(i,j)}_r$. We assume that the selection mechanism of response is ignorable, this is:

3 $$\begin{aligned} \pi ^{(j)}_k= P(\delta ^{(j)}_k = 1 | y_k, \textbf{x}_k) = P(\delta ^{(j)}_k = 1 | \textbf{x}_k); k \in s^{(j)}_r \, \end{aligned}$$

where $j=1,...,t$, and for $\delta ^{(i,j)}_k$:

4 $$\begin{aligned} \pi ^{(i,j)}_k= P(\delta ^{(i,j)}_k = 1 | y_k, \textbf{x}_k) = P(\delta ^{(i,j)}_k = 1 | \textbf{x}_k); k \in s^{(i,j)}_r \,. \end{aligned}$$

where $$i=1,...,j-1$$, $$j=2,...,t$$, and $$i<j$$.

We also assume that the mechanism follows a parametric model:

5 $$\begin{aligned} P(\delta ^{(j)}_k =1 | y_k, \textbf{x}_k) = m_{(j)} ( \textbf{x}_k, \lambda _{(j)}) \end{aligned}$$

and

6 $$\begin{aligned} P(\delta ^{(i,j)}_k =1 | y_k, \textbf{x}_k) = m_{(i,j)} ( \textbf{x}_k, \lambda _{(i,j)})\,. \end{aligned}$$

for some known functions $m_{(j)}(\cdot )$ and $m_{(i,j)}(\cdot )$ with second continuous derivatives with respect to unknown parameters $\lambda _{(j)}$ and $\lambda _{(i,j)}$, respectively. A commonly adopted parametric model is the logistic regression model [31, 32].

We use a state-of-the-art machine learning method: XGBoost [33] for estimating $\pi ^{(j)}_k$ and $\pi ^{(i,j)}_k$. This technique builds decision trees ensembles which optimize an objective function via Gradient Tree Boosting [34]. More details can be found in Annex 2. Kern et al. [12] has shown the effectiveness of this technique when studying non-response in the GSOEP panel. Ferri-García and Rueda [20] showed that Gradient Tree Boosting can lead to selection bias reductions in situations of high dimensionality or where the selection mechanism is Missing at Random (MAR). Lee et al. [35], Lee et al. [36], McCaffrey et al. [37], McCaffrey et al. [38], Tu [39], Zhu et al. [40], and Rueda et al. [41] have applied boosting algorithms in propensity score weighting showing better results than conventional parametric models.

In order to obtain the estimated propensities $\hat{\pi }^{(j)}_k$, we train a model with $s^{(j)}$ where $\textbf{x}_k$ includes every available variable observed in the BDLPA population framework, while to obtain the estimated propensities $\hat{\pi }^{(i,j)}_k$, we train a model with $s^{(i)}_r$ where $\textbf{x}_k$ includes every available variable observed in $s^{(i,j)}_r$. This model minimizes the weighted logistic loss for $\delta ^{(j)}_k ; k \in s^{(j)}$ and for $\delta ^{(i,j)}_k ; k \in s^{(i)}_r$.

Since the values we are interested in, $\hat{\pi }^{(j)}_k$ and $\hat{\pi }^{(i,j)}_k$ for $k \in s^{(j)}$ and $k \in s^{(i,j)}_r$, respectively, are a subset of the values used for training, $\delta ^{(j)}_k$ and $\delta ^{(i,j)}_k$ for $k \in s^{(j)}$ and $k \in s^{(i)}_r$, respectively, overfitting is likely to happen. This means that we will obtain values extremely close to 1 instead of real propensities. Hyperparameter optimization is essential in order to avoid this problem. This optimization can be applied as described in the Results section. Another important technique to consider is class balancing [42]. Classification models learn best when every class is equally represented in the training dataset. In practice, response rates are rarely close to 0.5 and therefore our model would often be biased. Class balancing ensures valid estimates, even when the response rate is high or low, by assigning ${(1 - p) \delta _k + p (1 - \delta _k)}$ as instance weight for training, where *p* represents the observed response rate. However, this method also distorts output probabilities. Consequently, they should be corrected as described by [43]:

$$\begin{aligned} \hat{\pi }_{corrected} = \frac{\hat{\pi } p}{\hat{\pi } p + (1 - \hat{\pi }) (1 - p) \,.} \end{aligned}$$

Then we applied this correction to the inverse of the estimated response propensity $\hat{\pi }^{(j)}_k$, which is ultimatley used as weight for constructing the estimator based on the new effective sample in $M_j$, $s^{(j)}_{r}$:

7 $$\begin{aligned} \hat{Y}^{\text {PSA}}_{s^{(j)}_{r}} = \sum _{k \in s^{(j)}_{r}} \frac{N}{n^{(j)}} \frac{n^{(j)}}{n^{(j)}_{r}} \frac{1}{ \hat{\pi }^{(j)}_{k}} y_{k}^{(j)} = \sum _{k \in s^{(j)}_{r}} d_{k}^{(j)} \frac{1}{ \hat{\pi }^{(j)}_{k}} y_{k}^{(j)} = \sum _{k \in s^{(j)}_{r}} d_{k}^{(j)PSA} y_{k}^{(j)} \,, \end{aligned}$$

where $j=1,...,t$; and we use the inverse of $\hat{\pi }^{(i,j)}_k$ as weight for constructing the estimator based on each effective sample of its corresponding panel $P_i$ created in the previous measurements until $M_j$, $s^{(i,j)}_{r}$

8 $$\begin{aligned} \hat{Y}^{\text {PSA}}_{s^{(i,j)}_{r}} = \sum _{k \in s^{(i,j)}_{r}} \frac{N}{n^{(i,j-1)}_{r}} \frac{n^{(i,j-1)}_{r}}{n^{(i,j)}_{r}} \frac{1}{ \hat{\pi }^{(i,j)}_{k}} y_{k}^{(i,j)} = \sum _{k \in s^{(i,j)}_{r}} d_{k}^{(i,j)} \frac{1}{ \hat{\pi }^{(i,j)}_{k}} y_{k}^{(i,j)} = \sum _{k \in s^{(i,j)}_{r}} d_{k}^{(i,j)PSA} y_{k}^{(i,j)} \,. \end{aligned}$$

where $$i=1,...,j-1$$, $$j=2,...,t$$, and $$i<j$$.

Combining these estimators, we can consider the following cross-sectional estimator for the total:

9 $$\begin{aligned} {} \hat{Y}^{\text {PSA}}_{M_j}= \sum _{i=1}^{j-1} \alpha _{i} \hat{Y}^{PSA}_{s^{(i,j)}_{r}} +\alpha _{j} \hat{Y}^{PSA}_{s^{(j)}_{r}} \,, \end{aligned}$$

where $j=1,...,t$ and $\alpha _{i}$ are nonnegative constants such that $\alpha _{1}+\alpha _{2}+...+\alpha _{j}=1$.

There are several ways to assign these constants. A simple solution is to weight each estimator by the weight that sample has in the total effective sample available at the time j. This permits the procedure not to depend on the variable to be estimated and also to calculate only a few $\alpha$ values, making the process of estimating the variables simpler and more systematic. This is the procedure we followed.

#### Calibration on population totals

In addition to modification of weights for handling non-response, it may also be carried out to take auxiliary information into account. Calibration [11] is the technique most used for weights adjustment and can aim to ensure consistency among estimates of different sample surveys, reduce biases in the sample due to non-response, non-coverage and other distortions, and also reduce variances [44–47].

Let $\textbf{x}^{*(j)}$ be a set of auxiliary variables related to *y* such that their population totals at the stratum level are known at measurement *j*, $\textbf{X}^{*(j)}_h= \sum _{\mathcal {U}_h}\textbf{x}^{*(j)}_{kh}$.

We denote by

$$\begin{aligned} \hat{Y}_{M_j}= \sum _{k \in s^{M_j}_{r} } D_{k}^{(j)} y_{k}^{(j)} \end{aligned}$$

any of the cross-sectional estimators obtained using the previous adjustment method, where $s^{M_j}_r = \cup _{i=1}^{j-1} s^{(i,j)}_r \cup s^{(j)}_r$, as we defined in the Sampling setup in overlapping panels section.

The calibration total estimator is obtained as:

10 $$\begin{aligned} \hat{Y}^{\text {CAL}}_{M_j}= \sum _{k \in s^{M_j}_r } w_k^{(j)} y_k^{(j)} \,, \end{aligned}$$

where the weights $w_k^{(j)}$, are as close as possible, with respect to a given distance *G*, to the weights $D_k^{(j)}$ obtained in the phase of reweighting and combination of samples:

11 $$\begin{aligned} \min _{\omega _{k}} \sum _{k \in s^{M_j}_r } {G\left( w_k^{(j)},D_k^{(j)}\right) } \end{aligned}$$

fulfilling the calibration condition

12 $$\begin{aligned} \sum _{k \in s^{M_j}_{rh} } w_{kh}^{(j)}\textbf{x}^{*(j)}_{jh} = \sum _{\mathcal {U}_h} \textbf{x}^{*(j)}_{kh}\ \end{aligned}$$

for all stratum *h* given by the calibration variables considered.

#### Estimating changes compared to the first measurement

A parameter of interest is the absolute change of a variable between one measurement and the first measurement. We denote by $\theta ^{\text {ABS}}_{M_j} = Y_{M_j} - Y_{M_1}$ this parameter, where $j=1,...,t$. Variations over time are measured more accurately with overlapping samples with respect to the case where samples on different occasions do not overlap (see [48]). An estimator of this parameter for measurement *j* based on the previous calibration total estimators can be obtained as follows:

13 $$\begin{aligned} \hat{\theta }^{\text {ABS}}_{M_j} = \hat{Y}^{\text {CAL}}_{M_j} - \hat{Y}^{\text {CAL}}_{M_1} \,. \end{aligned}$$

Another parameter of interest in panel surveys is the relative change $\theta ^{\text {REL}}_{Mj}= \frac{Y_{M_j} - Y_{M_1}}{ Y_{M_1}}$ between measurement 1 and measurement *j*, which is estimated as:

14 $$\begin{aligned} \hat{\theta }^{\text {REL}}_{Mj}= \frac{\hat{\theta }^{\text {ABS}}_{M_j}}{\hat{Y}^{\text {CAL}}_{M_1}} \,. \end{aligned}$$

The estimator is a quotient of two estimators of the total based on two different samples, meaning that its properties are not equivalent to those of the ratio estimator commonly used in survey sampling, but its theoretical properties can be derived by using Taylor linear approximation.

#### Estimating gender gaps in each measurement

The impact of COVID-19 on the social determinants of health may have differed significantly between women and men as shown in recent studies [49]. It is therefore of great interest to define the estimators of the gender gap observed in each measurement, and also in absolute and relative terms, in order to observe their evolution.

Let $Gen = \{M, W\}$ be the variable measured in $s^{(j)}, j = 1, ..., t$ which reflects whether a respondent is a man (*M*) or a woman (*W*). We define the two indicator variables: $I_{kh}^M= 1$ if the unit *k* in stratum *h* is a man and 0 elsewhere, and $I_{kh}^W$ in a similar way.

We start by defining the absolute gender gap estimator as follows:

15 $$\begin{aligned} \begin{array}{c} \widehat{GG}^{\text {ABS}}_{M_j} = \hat{Y}^{\text {CAL}W}_{M_j} - \hat{Y}^{\text {CAL}M}_{M_j} = \\ \\ = \sum _h \sum _{k \in s^{M_j}_{rh} } w_{kh}^{(j)} y_{kh}^{(j)} I_{kh}^W - \sum _h \sum _{k \in s^{M_j}_{rh} } w_{kh}^{(j)} y_{kh}^{(j)} I_{kh}^M . \end{array} \end{aligned}$$

This estimator is defined as the linear combination of two estimators in certain domains, hence its theoretical properties can be easily derived [48]. This estimator is the most simple one that can be built on the gender gap and can differentiate between men and women in measurement *j*. However, this estimator is subject to the base rate of each variable. For this reason, we define the relative gender gap estimator as follows:

16 $$\begin{aligned} \widehat{GG}^{\text {REL}}_{M_j} = \frac{\widehat{GG}^{\text {ABS}}_{M_j}}{\hat{Y}^{\text {CAL}M}_{M_j}} = \frac{\hat{Y}^{\text {CAL}W}_{M_j} - \hat{Y}^{\text {CAL}M}_{M_j}}{\hat{Y}^{\text {CAL}M}_{M_j}} \,. \end{aligned}$$

This estimator allows us to observe the gender gap in measurement *j* taking into account the base rate of the given target variable.

Thus, to obtain the cross-sectional estimator for the study variables of each ESSA measurement, we start from the H-T estimator (1) adjusted for non-response (7), combined from the panel and new samples (9) and finally calibrate to increase the representativeness of the sample (10). This estimator serves as the basis for calculating the absolute (13) and relative (14) change estimators between measurement *j* and 1, and for obtaining the different estimators to measure the absolute and relative gender gap in a given measurement (15 and 16).

### Longitudinal estimation

The primary objective of panel surveys is the production of longitudinal data series which are appropriate for studying the gross change in the population between collection dates, and for research on causal relationships among variables. To study these changes and understand their relationships, it is more convenient to use longitudinal samples than cross-sectional ones, since they reflect the variations of the variable in each individual and enable additional parameters to be estimated, such as the number of population individuals whose value of *y* increases, decreases or remains the same between a measurement and the previous one. The drawback of working with the longitudinal sample is that its size is smaller at each time and therefore the variance of the estimates can be large.

In this section, the previous estimated propensities for each unit *k* of sample $s^{(i,j)}_{r}$, $\hat{\pi }^{(i,j)}_{k}$, are used to reweight for non-response when estimating the absolute difference from $M_j$ to $M_{j-1}$ as:

17 $$\begin{aligned} \hat{Y}^{\text {PSA}}_{M_j - M_{j-1}} = \sum _{k \in s_r^{(M_j,M_{j-1})}} D_k^{(M_j,M_{j-1})} (y_{k}^{(j)} - y_{k}^{(j-1)}) \,. \end{aligned}$$

where $s_r^{(M_j,M_{j-1})} = \cup _{i=1}^{j-1} s^{(i,j)_{r}}$, and $j=2,...,t$. In this situation, the estimator is calculated by modelling the non-response of each panel $P_i$ created until $M_{j-1}$, that is, we estimate the propensities given by 6.

Thus, the estimated propensities for each unit *k* of the samples $s^{(i,j)}_{r}$, $\hat{\pi }^{(i,j)}_{k}$, are used in the first stage to reweight for adjusting non-response, obtaining the total estimator given by 17; and, in the second stage, calibration is applied to reweight these weights and obtain new ones, $v_{k}^{(j, j-1)}$, so as to obtain better population representativeness. The longitudinal estimator of the absolute difference can be defined as follows:

18 $$\begin{aligned} \hat{Y}^{\text {CAL}}_{M_j - M_{j-1}} = \sum _{k \in s_r^{(M_j,M_{j-1})}} v_k^{(j, j-1)} (y_k^{(j)} - y_k^{(j-1)}) \,. \end{aligned}$$

The longitudinal nature of the estimator allows us to define new estimators on the number of population individuals whose value of *y* increases, decreases or remains the same between $M_j$ and $M{j-1}$. Let *A* be a subset of interest ($\mathbb {R}^{+}$, $\mathbb {R}^{-}$ or 0 if we are interested in the units whose value of *y* increases, decreases or remains the same, respectively); the estimator of the number of population individuals for which $y^{(j)} - y^{(j-1)} \in A$ can be estimated as follows:

19 $$\begin{aligned} \hat{\theta }^{A}_{M_j-M_{j-1}} = \sum _{k \in s_r^{(M_j,M_{j-1})}} v_k^{(j, j-1)} I_A, I_A = \left\{ \begin{array}{rl} 1 &{} y_k^{(j)} - y_k^{(j-1)} \in A \\ 0 &{} y_k^{(j)} - y_k^{(j-1)} \notin A \end{array} \right. \,. \end{aligned}$$

We can also obtain the estimator of the rate of people whose value in *y* has decreased between $t-1$ and *t*, in reference to the people whose value in *y* has increased between $t-1$ and *t*. For example, if the variable *y* measures health status, this rate can be considered a deterioration/improvement rate, $\hat{\theta }^{\text {RATE}}_{M_j-M_{j-1}}$. The formula can be defined as follows:

20 $$\begin{aligned} \begin{array}{c} \hat{\theta }^{\text {RATE}}_{M_j-M_{j-1}} = \frac{\hat{\theta }^{A_{R^{-}}}_{M_j-M_{j-1}} - \hat{\theta }^{A_{R^+}}_{M_j-M_{j-1}}}{\hat{\theta }^{A_{R^+}}_{M_j-M_{j-1}}} = \\ \\ =\frac{\sum _{k \in s_r^{(M_j,M_{j-1})}} v_k^{(j, j-1)} I_{A_{R^-}} - \sum _{k \in s_r^{(M_j,M_{j-1})}} v_k^{(j, j-1)} I_{A_{R^+}} }{\sum _{k \in s_r^{(M_j,M_{j-1})}} v_k^{(j, j-1)} I_{A_{R^+}} }. \ \end{array} \end{aligned}$$

where

$$\begin{aligned} I_{A_{R^+}} = \left\{ \begin{array}{rl} 1 &{} y_k^{(j)} - y_k^{(j-1)} > 0 \\ 0 &{} y_k^{(j)} - y_k^{(j-1)} \le 0 \end{array} \right. \end{aligned}$$

and

$$\begin{aligned} I_{A_{R^-}} = \left\{ \begin{array}{rl} 1 &{} y_k^{(j)} - y_k^{(j-1)} < 0\\ 0 &{} y_k^{(j)} - y_k^{(j-1)} \ge 0 \end{array} \right. \end{aligned}$$

Based on both previous estimators, those based on the absolute and relative gender gap of the absolute difference between *j* and $j-1$ are defined as follows, respectively:

21 $$\begin{aligned} \begin{array}{c} \widehat{GG}^{\text {ABS}A}_{M_j - M_{j-1}} = \hat{\theta }^{A_{W}}_{M_j-M_{j-1}} - \hat{\theta }^{A_{M}}_{M_j-M_{j-1}} = \\ \\ =\sum _{k \in s_r^{(M_j,M_{j-1})}} v_k^{(j, j-1)} I_A I_k^W - \sum _{k \in s_r^{(M_j,M_{j-1})}} v_k^{(j, j-1)} I_A I_k^M \,, \end{array} \end{aligned}$$

22 $$\begin{aligned} \begin{array}{c} \widehat{GG}^{\text {REL}A}_{M_j - M_{j-1}} = \frac{\widehat{GG}^{\text {ABS}A}_{M_j - M_{j-1}}}{\hat{\theta }^{A_{M}}_{M_j-M_{j-1}}} \,. \end{array} \end{aligned}$$

A positive value of these estimators would indicate, in absolute (percentage points) or relative terms (percentages), that the percentage of women who improved/increased, remained the same, or deteriorated/decreased their outcome in the target difference variable was higher than the corresponding percentage in the male population, while a negative value would indicate that the percentage was lower in women. Estimator 21 is defined as the linear combination of two estimators in certain domains, while estimator 22 is a quotient of two estimators based on the same samples, hence their theoretical properties can be easily derived.

### Variance estimation

It is no simple task to develop suitable variance estimators for these proposed estimators taking into account the panel design used. The variance estimation problem in longitudinal surveys is addressed in several papers. For example, [28] considers variance estimation for Canada’s Survey of Labor and Income Dynamics within a Taylor linearization approach and a bootstrap method.

Some other works are developed for rotation panels: [17] considers the estimation of the variance of cross-sectional indicators for the SILC survey in Switzerland based on a four-panel rotation scheme where the non-response is modeled using a Poisson design. [50] considers variance estimation for weighting in the SILC survey in France with a rotation scheme consisting of four panels. Ardilly and Osier [31] considers the case of a panel survey in which solely the units in the original sample are followed over time, without reentry or late entry units at subsequent times to represent possible newborns. They assume a non-response model where the response probability at time *t* can be explained by the variables observed at times 0, $t-1$, including the variables of interest. Zhou and Kim [51] also consider the estimation of a mean for a panel survey, in case of monotone non-response.

On the other hand, there is little work about variance estimation for machine learning methods. Some work about variance estimation for tree-based methods is the infinitesimal jackknife [52].

In this study, the formulas used for estimating the variance of indicators must take into account the structure and complexity of the ESSA survey. The main factors to consider for estimating the variance of the proposed estimators are the non-linearity of the estimators, total non-response at different survey stages and the use of machine learning models in conjunction with calibration. Therefore, we consider the application of bias-corrected and accelerated bootstrap [53]. It is well suited for a wide variety of scenarios, including ours, and it is easy to efficiently implement via Scipy [54], a standard Python scientific library.

## Results

### Observed non-response biases

**Fig. 2:**
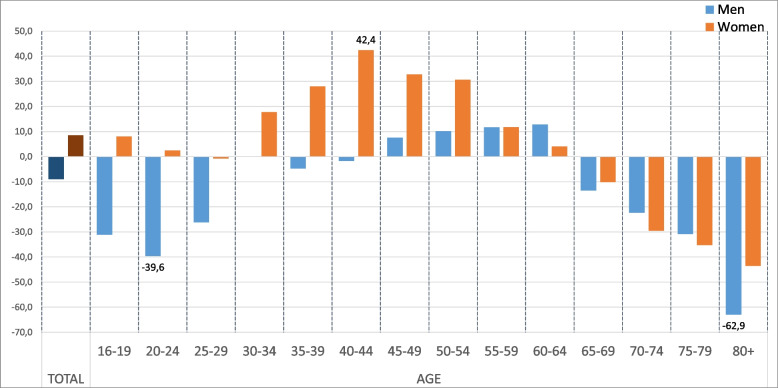
Observed biases for the calibration variables in measurement 4 (age and sex)

**Fig. 3:**
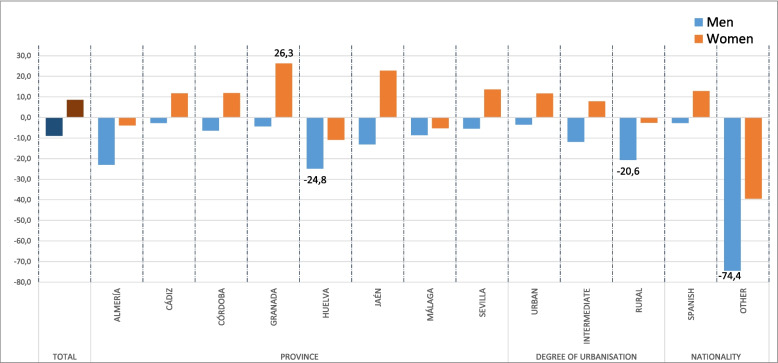
Observed biases for the calibration variables in measurement 4 (sex-province, sex-urbanization and sex-nationality)

To illustrate the observed biases produced mainly by non-response in the ESSA survey, Figs. 2 and 3 show the differences between the sample and the study population at measurement 4. These differences are according to the intersection of the sex variable with age, province, degree of urbanization and nationality. Thus, with respect to age, the largest differences between the values observed from the sample and those from the population are found in the youngest men (under 30 years old), in middle-aged women (between 35 and 54 years old) and in the oldest women and men (over 70 years old), these differences increasing with age. With regard to the other segmentation variables, the largest differences were found among people with a nationality other than Spanish, especially among men. These results are also observed although to a lesser extent in the previous measurements, showing a lower participation of these population groups in the ESSA, and therefore justify the need to adjust the sample weights.

### Modelling non-response

ESSA thus has a non-monotone missing pattern and shows a lower participation of some population groups. This non-response, given in the new theoretical samples created in each measurement and in the panels involved in each measurement, is modelled with PSA as explained in the Methods section. In order to ensure that the XGBoost model is learning properly, we considered the following hyper-parameters:

- Number of estimators $\in [10, 1000]$: the number of trees forming the ensemble.
- Learning rate $\in [0.001, 0.9]$: the weight shrinkage applied after each boosting step.
- Maximum depth $\in [1, 30]$: the maximum number of splits that each tree can contain.
- Minimum child weight $\in [0, 10]$: the minimum total of instance weights needed to consider a new partition.
- Subsample $\in [0.6, 1]$: proportion of training data which is randomly sampled for each iteration.

The accuracy of the algorithm was tested with cross-validation. Therefore, training data is partitioned into 5 complementary subsets so that each one has the same proportion of $\delta ^{(t)}_k = 1$ and $\delta ^{(t)}_k = 0$ as the total. Then 5 models are trained leaving each one of the subsets out of the training data. For each model, the logistic loss was calculated for its corresponding remaining subset. The mean logistic loss is the estimated error.

The values for the hyperparameters minimizing this estimated error were obtained using the Tree-structured Parzen Estimator (TPE) algorithm [55, 56]. TPE is implemented as default method in Optuna [57], an optimization library for Python.

The cross-sectional and longitudinal estimators were calculated by using these PSA weights.

### Calibrating sample representativeness

As explained in the Methods section, the weights obtained to adjust non-response are reweighted by calibration to achieve better representativeness of the population and reduce biases in the cross-sectional and longitudinal estimators.

The first ESSA measurement was carried out by IECA as another edition of the Social Household Survey that they have been conducting since 2007. Similarly, to deal with the observed biases, we had to apply the same adjustment as IECA for the sample weights of the new samples and panels of each ESSA measurement, i.e. truncated raking calibration and the total population size for the intersection of the sex variable with province, age, urbanization grades and nationality as auxiliary information. The data for these totals were obtained from the 2019 Municipal Register of Inhabitants [58].

### Cross-sectional estimators

**Fig. 4:**
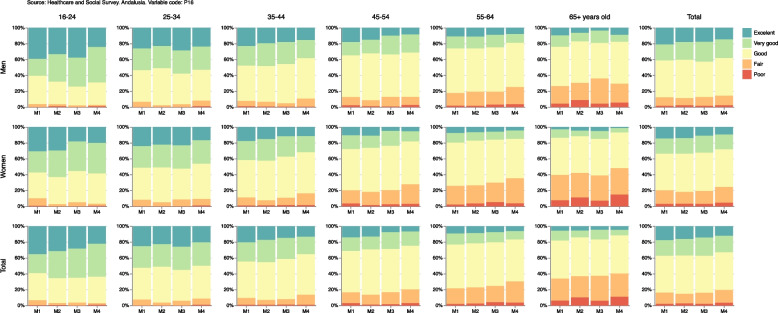
Estimations grouped by sex and age for the original categories of self-perceived general health

Supplementary Table 1 shows for measurement 4 the percentages with corresponding 95% confidence intervals in addition to the sample size for each original category of the self-perceived general health variable grouped by sex and age. It may be observed from the chart that the percentages for the ‘excellent’ or ‘very good’ categories do not follow a clear pattern throughout measurements for the population between 16 and 34 years old, and 65+, either for men or for women. However, ‘excellent’ or ‘very good’ self-perceived health decreases for the population between 35 and 64 years old as the pandemic advances. This can be observed more as age increases, especially in women. This reduction results in an increment for the ’fair’ and ’bad’ categories. However, the ’good’ general health category remains stable throughout the pandemic for each sex and age group. Figure 4 shows the percentages and confidence intervals given in Supplementary Table 1 not only for measurement 4, but also for all other ESSA measurements.

**Fig. 5:**
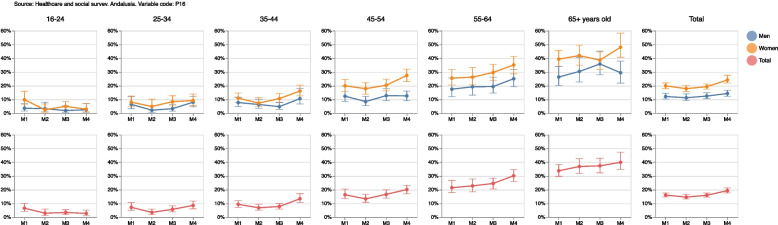
Percentages and confidence intervals at 95% level of people with fair or poor self-perceived general health

Based on these results, we dichotomized this variable with the categories ’excellent, very good and good’ and ’fair and poor’. For each ESSA measurement, Supplementary Table 2 shows the percentages and 95% confidence intervals of this dichotomized self-perceived general health variable. These results can be seen in Fig. 5, which shows an increase in ’fair and poor’ self-perceived health in measurements 3 and 4, this increase being slightly larger among women. Regarding age groups, evolution remained stable throughout the pandemic from lockdown onwards for the population aged between 16 and 24 for men and women alike. However, for the population aged over 25, the evolution worsens as age increases and the pandemic advances, especially in women. Therefore, this subpopulation got the highest ’fair or poor’ general health values from the beginning of the lockdown for every age group above 25 years old.

**Fig. 6:**
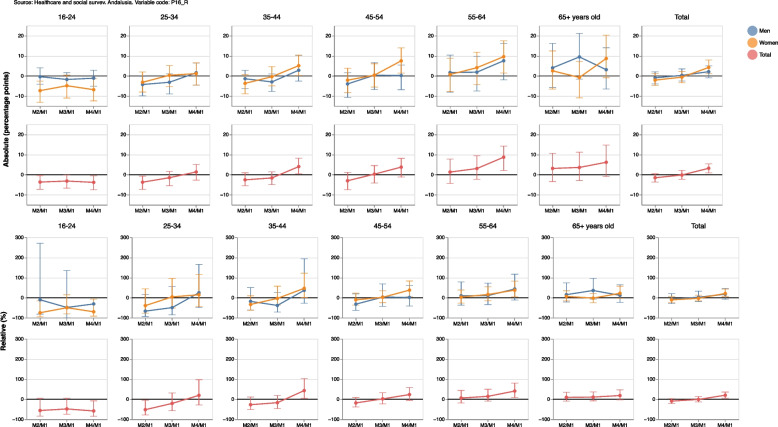
Absolute percentage changes and 95% confidence intervals for people with fair or poor self-perceived general health

Supplementary Table 3 shows the relative percentage changes and 95% confidence intervals for each measurement compared to measurement 1 for the ’fair or poor’ self-perceived general health variable, while Fig. 6 shows the absolute percentage changes. It can be seen that fair or poor perception of general health increased in the general population by a 20% (CI95%=[5.2; 35.7] in measurement 4 compared to measurement 1. This increase was observed in all age groups, except for people under the age of 24 and over 65 years old, with no differences between women and men.

**Fig. 7:**
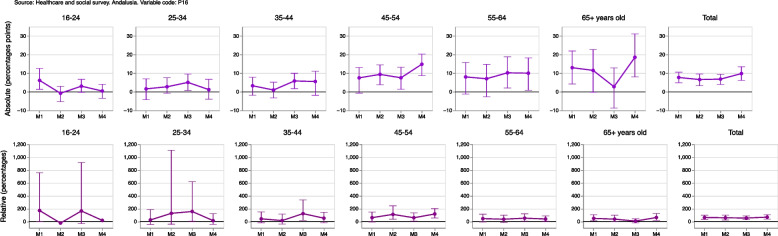
Absolute and relative gender gap for the change in the fair or poor self-perceived health in each measurement (M)

Supplementary Table 2 also includes absolute and relative gender gaps, i.e. the absolute difference (in percentage points) and the relative difference (in percentages) between women and men of a given measurement compared to the first measurement in which the target variable was gathered. This can be interpretated as a positive value indicating that women showed a positive difference (absolute or relative) in comparison to men in their ’fair or poor’ self-perceived general health. This result could therefore be seen as a negative gender gap in the corresponding measurement (i.e. worse result or unfavorable to women as the reference category is ’fair or poor’). By contrast, a negative value would indicate that women showed a negative difference (absolute or relative) in comparison to men in their ’fair or poor’ self-perceived general health, which could be seen as a positive gender gap (i.e. better result or favorable to women). These results are shown in Fig. 7; we can see, for example that both the absolute and relative gender gaps were positive throughout the pandemic, confirming an increasingly negative impact on women compared to men in terms of fair or poor self-perceived general health. Results by age reveal that the largest positive gender gaps were observed in people over 45 years old.

### Longitudinal estimators

**Fig. 8:**
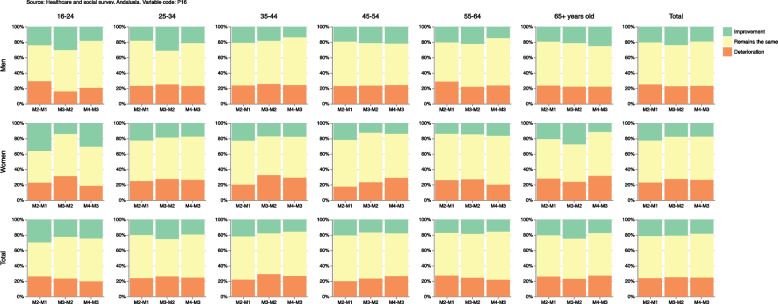
Percentage of population whose self-perceived general health improves, deteriorates or remains the same

Supplementary Table 4 shows estimates of better, equal or worse self-perception of health in the population for a given measurement compared to the same population in the previous measurement. Thus, 21.7% of the study population improved their self-perceived general health in measurement 2 compared to measurement 1, but this percentage was slightly smaller in subsequent measurements. By contrast, 23.8% of this population group presented worse self-perceived general health in measurement 2 compared to measurement 1, with this percentage being slightly higher in subsequent measurements. When we analyze these results by sex and age, it can be observed that it is women between 25 and 54 years old who experience the decreases in the improvement of general health over the course of the pandemic and, conversely, women between 45-54 years old who experience an increase in the deterioration of self-perceived general health. On the other hand, the percentage of people who had remained the same self-perceived general health status in a given measurement compared to the previous one did not vary over the course of the pandemic, except for the population below 24 years old which did experience increases in the aforementioned percentage, going from 44% in measurement 2 to 55.9% in measurement 4. These results are shown in Fig. 8.

**Fig. 9:**
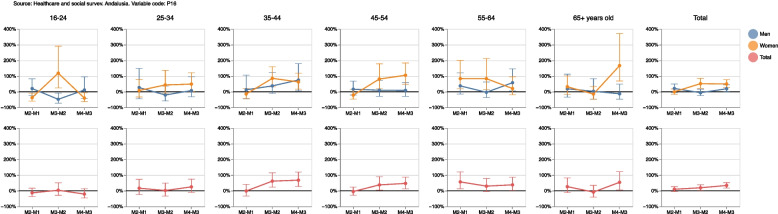
Population whose health worsens between one measurement and the previous one compared to the population whose health improves

If we calculate the ratio of the population with worsening self-perceived general health (in a given measurement compared to the previous one) and the population where it improves, a positive value means that there are more people whose self-perceived general health has deteriorated than people whose health has improved, as seen in Fig. 9. In relative terms it could be observed that, in measurement 2 compared to measurement 1, 10% more of the population had worse self-perceived health than better health; this percentage increased to 19.5% and 34.2% in measurements 3 and 4 compared to measurements 2 and 3, respectively. These differences are larger in women, reaching values of 51.9% and 49.8% in measurements 3 and 4, respectively. If the ratio is analyzed according to the age of individuals regarding measurement 3 compared to measurement 2, deterioration of health was more frequently observed in women of any age.

**Fig. 10:**
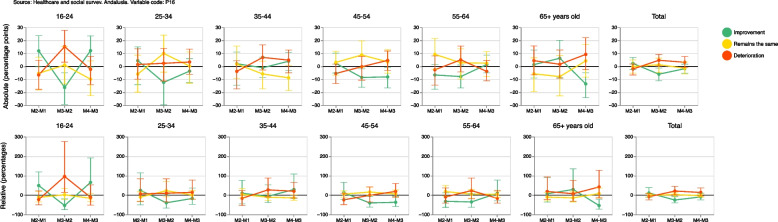
Absolute and relative gender gaps in the improved, equal or worse self-perceived general health

Supplementary Table 4 also shows absolute and relative gender gaps in improvement self-perceived general health, staying the same or deteriorating in a measurement compared to the previous one in the same population. On the one hand, absolute gender gap is the absolute difference (in percentage points) between women and men with better, equal or worse self-perceived health in a measurement compared to the previous one, and on the other hand relative gender gap is the relative difference (in percentage) between women and men with better, equal or worse self-perceived health. This means that a positive value in the gap (absolute or relative) indicates that the percentage of the female population with improved, equal or worsened self-perceived general health was greater than the corresponding percentage in the male population. A negative value would indicate that the percentage was smaller in women. Regarding deterioration of health, we observe in Fig. 10 that the percentage of the female population whose self-perceived health was worse in measurement 2 than in measurement 1 was 8.2% lower than among their male counterpart. However, this relative gender gap in health deterioration became positive in subsequent measurements, increasing to 20.9% and 13.8%, i.e. the deteriorating percentages were greater among women in measurement 3 and in measurement 4. This result was observed across all age groups, except for the population younger than 24 years old and the population between 55 and 64 years old.

Tables 1 and 2 summarize the name, table, figure, formula and interpretation relating to the estimators developed throughout this paper for cross-sectional and longitudinal samples, respectively.

**Table 1:** Name, table, figure, formula and interpretation of each estimator developed for the cross-sectional samples

NAME	TABLE	FIGURE	FORMULA	INTERPRETATION
Original variables	1	4	(10)	Percentages, confidence intervals at 95%, sample size and population estimations at measurement 4, grouped by sex and age, for the original categories of self-perceived general health.
Dichotomized variables	2	5	(10)	Evolution of percentages and confidence intervals at 95%, grouped by sex and age, of people with fair or poor self-perceived general health. If the confidence intervals for the same measurement do not overlap, it can be said that there are statistically significant differences between women and men. Similarly, if the confidence intervals of two different measurements do not overlap, it can be said that there are statistically significant differences between them.
Absolute/Relative change	No/3	6	(13)/(14)	Evolution of absolute/relative changes and confidence intervals at 95%, grouped by sex and age, of people with fair or poor self-perceived general health in each measurement compared to measurement 1. A positive value indicates an increase, in percentage points/terms, in the fair or poor self-perception of overall health of the corresponding measure compared to the first measure. Conversely, a negative value indicates a decrease, in percentage points/terms, in the fair or poor self-perception of overall health of the corresponding measure compared to the first one. If the confidence interval does not include the value 0, this increase or decrease can be said to be statistically significant. Similarly, if the confidence intervals for the same measurement do not overlap, it can be said that there are statistically significant differences between women and men.
Absolute/Relative gender gap	2	7	(15)/(16)	Evolution in each measurement (M) of absolute/relative gender gaps (women versus men) and confidence intervals at 95%, grouped by age, of people with fair or poor self-perceived general health. A positive value indicates that women show, in percentage points/terms, a larger value in comparison to men in their ’fair or poor’ self-perceived general health of the corresponding measurement. Therefore, this result could be seen as a negative gender gap (i.e., worse result or unfavorable to women) in the corresponding measurement. Conversely, a negative value indicates that women showed, in percentage points/terms, a smaller value in comparison to men in their ’fair or poor’ self-perceived general health. It could be seen as a positive gender gap (i.e., better result or favorable to women) in the corresponding measurement. If the confidence interval does not include the value 0, the corresponding gender gap can be said to be statistically significant.

**Table 2:** Name, table, figure, formula and interpretation of each estimator developed for the longitudinal samples

NAME	TABLE	FIGURE	FORMULA	INTERPRETATION
Longitudinal difference	4	8	(19)	Percentage of population and confidence intervals at 95% whose self-perceived general health increases/improves, decreases/deteriorates or remains the same between a measurement and the previous one
Decrease Increase Rate	No	9	(20)	Percentage of the population and confidence intervals at 95% that worsens their general health (in a given measurement compared to the previous one) and the population that improves it. A positive value means that there are more people whose self-perceived general health has deteriorated than people whose health has improved.
Absolute/Relative gender gap in the absolute difference	4	10	(21)/(22)	Absolute/Relative difference (in percentage points/terms) and confidence intervals at 95% between women and men with better, equal or worse self-perceived health in a measurement compared to the previous one. A positive value indicates that the percentage of the female population with improved, equal or worsened self-perceived general health was greater than the corresponding percentage in the male population. A negative value would indicate that the percentage was smaller in women.

## Discussion

The rapid evolution of the COVID-19 pandemic has forced researchers to provide timely estimates on the disease’s impact on the population. This has often led to the creation of survey studies which did not meet the criteria for being considered probabilistic, entailing many sources of error that may affect the final estimates obtained from them. In this sense, a recent scoping review on the methodological characteristics of the health surveys conducted in Spain early on in the COVID-19 pandemic included 55 studies (among over 3000 initially identified) [4]. An outcome of this review worth noting is the low proportion of longitudinal surveys identified (12.7%) and the implementation of some type of sampling adjustment (30.9%), even though most of the surveys were based on non-probability sampling (92.7%). Moreover, none of them considered the ESSA design or the reweighting approach described in this paper. Therefore, the ESSA survey is particularly valuable in the sense that its overlapping probability panel design offers the opportunity to obtain reliable estimates, both cross-sectional and longitudinal, on the impact of COVID-19 on health and its determinants. However, the analysis of the survey has not been exempt from statistical adjustments to correct for attrition and survey non-response.

The two-step adjustment procedure has been established in this study to remove the two main sources of error in the sampling design: population non-response, understood as people who did not take part in the survey despite having been selected in the sample, which was treated in the calibration step, and panel non-response, understood as people who participated in some of the measurements but did not follow up in subsequent ones. Panel non-response has been treated using PSA, which is a technique often used for addressing selection bias in online surveys [59] but which can also be used for non-response; in fact, it was originally adapted from [29] for this matter [60].

In our study, the XGBoost technique has been used to model lack of response from one measurement to another. The authors in [21] propose a novel neural network structure and they compare it with advanced gradient boosting methods such as XGBoost, using a justification that these are still state-of-the-art in spite of their age. It would be of great interest to consider their recent proposal in order to model non-response. However, it is still very experimental, as evidenced by the scarcity of papers and implementations. For such an important application as the ESSA, we therefore prefer an established method. The application developed in this work is one example where techniques in the machine learning field need to be combined with other important techniques in survey research, such as calibration and PSA, when studying non-response in a panel setting.

The propensities obtained could also be further analyzed by interpretable models [61] in order to determine the factors associated with non-response. This would help establish strategies for obtaining higher response rates, or at least for offsetting low response rates by targeting specifically hard-to-reach groups.

The results observed in the different estimates obtained from the self-perceived general health variable show that the impact of the pandemic has affected age age groups and genders differently. More precisely, self-perceived general health seems to have decreased more notably in older age groups and among women, according to the evolution of cross-sectional estimates and longitudinal estimates alike. The gender gap in both absolute and relative terms generally increased as the pandemic advanced, meaning that the differences (mostly decreases in self-perceived general health) have been larger and worse in women than in men. We chose this important health outcome because of the enormous amount of research it invests in studying risk factors and policy interventions in Public Health. This is due to its ability to summarize more objective measures such as morbidity, mortality, and clinical assessments of health conditions [62].

Some limitations must be noted in this study. Firstly, it is a well-known fact that subjective variables usually entail measurement errors, as the response given in such questions by the interviewee may depend on numerous unmeasurable factors unrelated to the subject being studied, but which distance the final response from the objective value that should be given. Further studies should consider the measurement of such variables using validated instruments for a more objective understanding of the subject. In any case, the methodology developed in this research can be extended to any variables and scales.

Secondly, we assume a covariate-dependent missingness pattern, as is usual in propensity score adjustment [63–65]. In a panel survey, it may be more realistic to assume Missing at Random which allows for dependence on the observed *y*-values in the previous years [31, 51], but has the drawback of the adjustment weights varying for each variable, which is not useful for multipurpose surveys such as the ESSA. This survey has more than 400 variables and is used by health researchers from different specialties, the objective being to give adjusted weights to each unit of the sample so that each researcher can use them to carry out their specific studies related to the variables that interest them. It would also be interesting to see the differences between the estimates with these two different patterns and whether this difference in accuracy offsets the complexity of having to build a different response model for each variable.

Another limitation in this work is that we have considered a situation in which the study population does not vary over time. This is justified because the new measurements are made with little difference compared to the first measurement (at one month, 6 months and 12 months) and all the samples are obtained from the same sampling frame [23], so we have assumed that the sample designs refer to the same population. In fact, the difference in population between the 2019 and 2020 population frameworks is 0.6% in relative terms, or, in absolute terms, about 42,000 people out of almost 7.2M people over 16 years of age residing in Andalusia [23].

These methods would therefore not be well suited to overlapping panel surveys where samples are drawn from very different frames in different years and therefore from different populations. In such cases, the proposed methodology would have to be adapted.

## Conclusion

For addressing future health crises such as COVID-19, potential coverage and non-response biases in surveys must be reduced by means of utilizing reweighting techniques.

In this respect, we propose a new reweighting approach to produce suitable estimators for both cross-sectional and longitudinal samples in overlapping panel surveys. To achieve this, first the original sampling design weights are corrected by modelling non-response in respect of the longitudinal sample obtained in a previous measurement using machine learning techniques, and then, they are calibrated using the auxiliary information available at the population level.

We apply this methodology to estimate totals, proportions, ratios, and differences between measurements as well as gender gaps in the variable of self-perceived general health. The descriptive results for this variable are an example applied to this paper to show the different estimators, tables and figures developed which can be replicated with other variables and scales from other overlapping panel surveys. In fact, they are all extended to the 400+ ESSA variables through the web platform at www.easp.es/info/ESSA. On this website, after selecting the set of variables to be described, the estimators to be shown and the segmentation variables to be considered (sex and age or sex and degree of urbanization), the user obtains the corresponding interactive figures to help interpret the selected variables. This will allow the scientific epidemiological research community not only to access the descriptive results for all the ESSA variables, but also to carry out their own analyses by downloading the ESSA database and code developed, used as the basis for the conclusions of this paper.

### Supplementary Information

**Additional file 1: Table 1.** Estimations grouped by sex and age for the original categories of self-perceived general health at measurement 4 (Excel file).

**Additional file 2: Table 2.** Percentages, gender gaps and confidence intervals at 95% of people with fair or poor self-perceived general health (Excel file).

**Additional file 3: Table 3.** Relative percentage changes and 95% confidence intervals for people with fair or poor self-perceived general health (Excel file).

**Additional file 4: Table 4.** Percentage of people whose self-perceived general health improves, deteriorates or remains the same, and absolute and relative gender gap (Excel file).

## Data Availability

The dataset and code supporting the conclusions of this article is available in the ESSA repository at www.easp.es/info/ESSA.

## Abbreviation

ESSA: Spanish acronym for Encuesta Sanitaria y Social de Andalucía (Andalusia Healthcare and Social Survey)
